# A Low Energy Approach for the Preparation of Nano-Emulsions with a High Citral-Content Essential Oil

**DOI:** 10.3390/molecules26123666

**Published:** 2021-06-16

**Authors:** Suelen F. Pereira, Adenilson Barroso, Rosa H. V. Mourão, Caio P. Fernandes

**Affiliations:** 1 Post-Graduate Program in Pharmaceutical Innovation, Federal University of Amapá, Macapá 68903419, Amapá, Brazil; sufelixbio@gmail.com; 2 University of the State of Amapá, Macapá 68903419, Amapá, Brazil; 3 Laboratory of Phytopharmaceutical Nanobiotechnology, Federal University of Amapá, Macapá 68903419, Amapá, Brazil; 4 Laboratory of Bioprospection and Experimental Biology, Oeste do Pará Federal University, Santarém 68040070, Pará, Brazil; adenilson.barroso@yahoo.com.br (A.B.); mouraorhv@yahoo.com.br (R.H.V.M.); 5 Bionorte Post-Graduate Program (Network Program)–Rede de Biodiversidade e Biotecnologia da Amazônia Legal, Oeste do Pará Federal University (Local Pole), Santarém 68040070, Pará, Brazil

**Keywords:** citral ratio, colloids, dynamic light scattering, droplet variation, monoterpenes

## Abstract

*Pectis elongata* is found in the northern and northeastern regions of Brazil. It is considered a lemongrass due to its citric scent. The remarkable citral content and the wide antimicrobial properties and bioactive features of this terpene make this essential oil (EO) eligible for several industrial purposes, especially in cosmetics and phytotherapics. However, to address the problems regarding citral solubility, nano-emulsification is considered a promising strategy thanks to its improved dispersability. Thus, in this paper we propose a low-energy approach for the development of citral-based nano-emulsions prepared with *P. elongata* EO. The plant was hydrodistillated to produce the EO, which was characterized with a gas chromatograph coupled to mass spectrometry. The nano-emulsion prepared by a non-heated water titrating (low-energy) method was composed of 5% (*w*/*w*) EO, 5% (*w*/*w*) non-ionic surfactants and 90% (*w*/*w*) deionized water and was analyzed by dynamic light scattering. Levels of citral of around 90% (neral:geranial—4:5) were detected in the EO and no major alteration in the ratio of citral was observed after the nano-emulsification. The nano-emulsion was stable until the 14th day (size around 115 nm and polydispersity index around 0.2) and no major alteration in droplet size was observed within 30 days of storage. Understanding the droplet size distribution as a function of time and correlating it to concepts of compositional ripening, as opposing forces to the conventional Ostwald ripening destabilization mechanism, may open interesting approaches for further industrial application of novel, low-energy, ecofriendly approaches to high citral essential oil-based nano-emulsions based on lemongrass plants.

## 1. Introduction

Some species from the genus *Pectis* are commonly known as lemongrasses due to the citric scent of their volatiles, which are similar to *Cymbopogon citratus* [1,2]. In fact, a literature review showed that, due to this unique phytochemical profile, *Pectis* spp. has been used as substitutes to replace the true *C. citratus* lemongrass. The wide range of ethnopharmacological applications of the tea and infusion preparations include the treatment of colds, grippes and fever, anxiety, hypertension, stomach disorders and hypotension and use as a calmative [2]. However, despite this, the essential oils (EOs) obtained from the species of this genus show a high amount of monoterpenes that may be mostly represented by citral; in some cases, high amounts of other monoterpenes, such as α-pinene and limonene, may be observed, impairing the scent [3,4].

*Pectis elongata* is a species found in the northern and northeastern regions of Brazil, and the tea of its aerial parts is commonly used in these regions to treat fever [5] and hypotension and as a calmative. It is also used as a replacement of citronella by the Creole population of French Guiana [3]. One of the first phytochemical studies with the species revealed a predominance of citral in its bacteriostatic and fungistatic EO [6]. However, despite studies about this species being scarce, indications that two chemotypes—citral (neral- and geranial-rich EO) and perillal (limonene-rich EO)—occur have been reported [3].

Citral is an aldehyde terpene constituted by a mixture of two structural isomers (Z-citral or neral and E-citral or geranial) [7] and citral-based plants are potential raw materials or sources of this terpene for cosmetic formulations, such perfumes, creams, lotions or shampoos [8].

One might expect that the bioactive properties of citral (e.g., anti-inflammatory action) [9] would be of interest for their uses in several novel industrial products. However, the EOs have intrinsic characteristics that impair the fulfillment of their full industrial potential, such as hydrophobicity, volatility and chemically instability. Thus, one of the main technological challenges is to maintain the original desirable characteristics in EO-based systems.

Nanodelivery systems can successfully overcome this issue and are a promising approach to maintain and also improve the quality, safety and functionality of EO-based products [10], allowing the obtainment of high-value nanophytopharmaceuticals directly linked to industrial crops, such as those rich in EOs. In this context, special attention should be given to oil in water nano-emulsion, since it can disperse the EOs as fine nanodroplets in aqueous media. Two main approaches have been used in the generation of nano-emulsions: high-energy and low-energy methods. The first group makes use of highly disruptive forces generated by specific devices. These devices include ultrasound generators. Double nano-emulsions containing glycyrrhizic acid, a sesquiterpene saponin, were obtained with this technique using skim milk as the external phase of this W/O/W colloid [11]. However, the high-energy methods elevate the costs of the process for industrial applications, since the high-scale equipment is more expensive compared to conventional method and, furthermore, most of the energy used in the nano-emulsification is lost in the process. The second group makes use of the chemical energy of the system to generate the nanodroplets and, if well designed, these nano-emulsions can also be scalable [12]. Different materials can be used to improve the stability of a colloid, often by forming a steric layer around the internal phase. Nowadays, biodegradable adjuvants are expected and, for example, mucilage from *Opuntia ficus indica* has been considered promising [13].

In the context of low-energy methods, if the nano-emulsifier is a surfactant it can trigger some changes that may sub-divide this main group according to the involved phenomena. If there is no change in the spontaneous curvature of the surfactant (or if no surfactant is used) and the nano-emulsification occurs due to a rapid diffusion of hydrophilic molecules to the external phase, the process is called spontaneous nano-emulsification. However, if there is a change in the spontaneous curvature of the surfactant during the generation of the nano-emulsions, phase inversion temperature (PIT) or phase inversion composition (PIC) methods can be used [12]. The latter are of great interest for EOs and, by using specific blends of hydrophilic–lipophilic biodegradable surfactants, it is possible to achieve stable and promising EO-based oil in water nano-emulsions. As part of our ongoing studies with these natural volatile oils, we have obtained optimal nano-emulsions with EOs from different species using ecofriendly, low-energy methods with a view to their further practical applications [14,15]

Therefore, we hypothesized that nano-emulsions containing this lemongrass (*Pectis elongata*) EO rich in citral can be generated with a non-heating, low-energy method and that the process would not affect the profile of the EO nor the citral ratio after the nano-emulsification.

## 2. Results

### 2.1. Essential Oil

The hydrodistillation yielded 1.52% of the EO of *P. elongata*. The main compounds (Table 1) were geranial (**1**) (50.76%) and neral (**2**) (39.35%), accounting for a citral content corresponding to around 90% of the total relative composition. The third main compound (isocitral<E->) (**3**) corresponded to only 1.41%of the EO.

### 2.2. Nano-Emulsification and Citral Ratio

**Figure 1:**
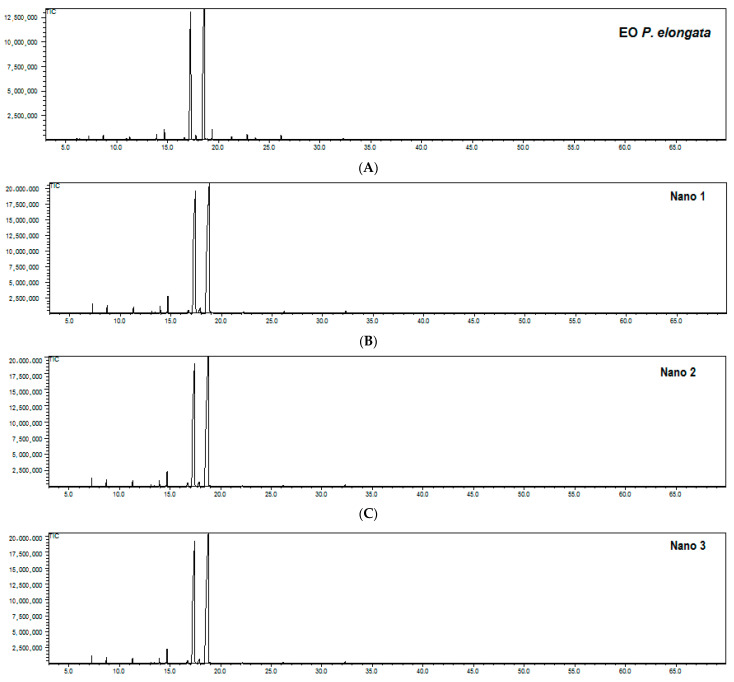
Total ion chromatograms of—from (**A**–**D**)—the *Pectis elongata* essential oil and the three replicates of its nano-emulsions.

Figure 1 shows the chromatographic profile of the EO extracted with the *P. elongata*-based nano-emulsions immediately after the process of its preparation (Figure 1B–D). All replicates were similar to the EO (Figure 1A), indicating that the nano-emulsification did not affect the overall phytochemical profile of the EO. The CRNE was 1.22 ± 0.03 and corresponded to 94.56% of the CROE, which also indicated that the ratio between the geranial and neral compounds, the two main monoterpenes of the EO, was not altered after the nano-emulsification.

Another important parameter in the development of nano-emulsions is the surfactant choice. In the present study, the HLB value blend of the surfactant (constituted by polysorbate 80 and sorbitan trioleate) was 13 and allowed a droplet size of around 116 nm.

### 2.3. Physical Characterization and Nanodroplet Behavior

**Figure 2:**
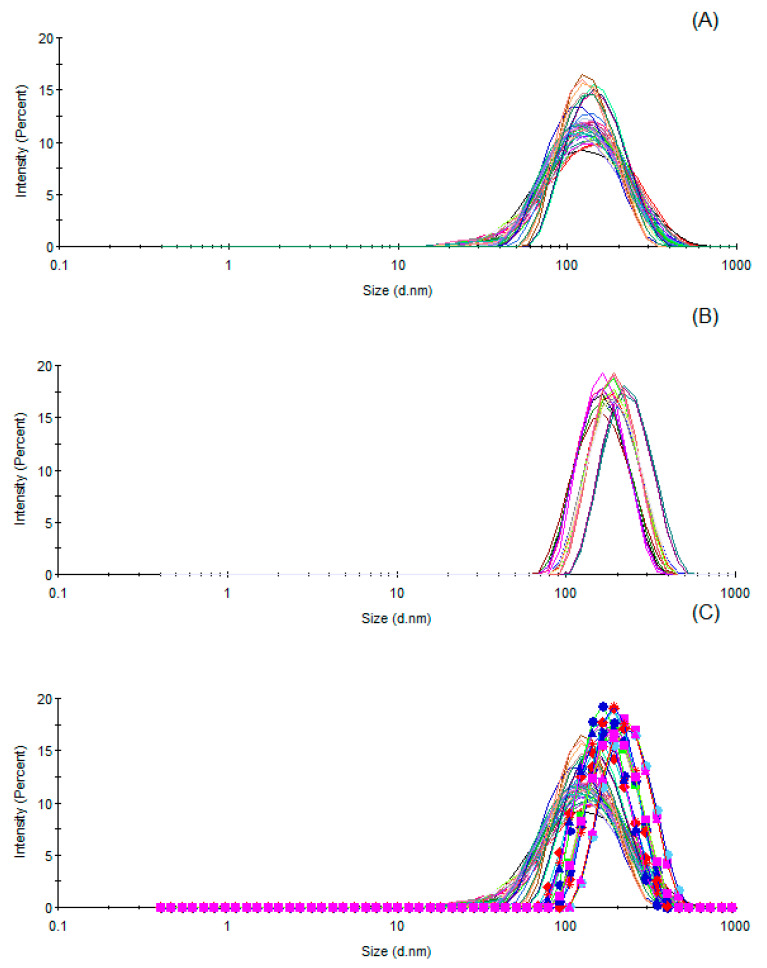
Droplet size distribution graphs of the Pectis elongata-based nano-emulsion. Superimposed graphs show measurements after (**A**) 0–30 days of storage, (**B**) 60–90 days of storage and (**C**) 0–90 days of storage. Colors representing size distribution curves of three independent batches of the prepared nano-emulsions. Measurements were performed in triplicate.

Table 2 shows the size, PdI and zeta potential values of the nano-emulsion prepared with the EO from *P. elongata*. During the first 14 days of storage (up to day 14), no major alterations in the size diameter were observed. Then, a slight tendency for droplet growth was observed up to 30 days of storage (DG_14,30_ = 13.88%). Despite the droplets increasing growth up to 90 days of storage (DG_30,60_ = 26.82%, DG_60,90_ = 18.52%), the PdI presented an overall decreasing tendency and stabilized between 30 and 90 days of storage after reaching a minimum (<0.1). Low values (in module) of the zeta potential were observed, probably due to the low polarizability of the EO components and utilization of non-ionic surfactants. Figure 2 shows the droplet size distribution graphs, in term of intensity, of the nano-emulsions. It is possible to see the narrowing of the curves as a function of the time of storage. This behavior was more evident in the period between 60 and 90 days of storage (Figure 2B), as observed by the displacement of the curves to slightly higher diameter sizes. It is interesting to note that citral (geranial and neral) showed a low logP (<4.3) value (3.17) and high water solubility (84.71 mg/L), as observed using the Chemspider Platform. These properties can been used to explain the behavior of EO-based nano-emulsions.

## 3. Discussion

The essential oil content was in accordance with the citral chemotype of the plant and corroborates its denomination as lemongrass [1,2].

Low-energy methods claim to make use of the chemical energy of a system to generate the fine droplets instead of using high disruptive forces as in the case of high-energy methods [12]. Despite the fact that some nano-emulsions were successfully generated with a low-energy method involving a heating step—which one would expect to be in accordance with changes in the spontaneous curvature of the non-ionic surfactant associated with the phase inversion temperature method [16]—we decided that this approach should be avoided, due to the intrinsically volatile nature of the EO compounds, and replaced by methods that work in accordance with phase inversion composition concepts [17,18]. However, to the best of our knowledge, most of the studies in the literature do not investigate whether the low-energy non-heating method properly maintains the profile of the EO after the process of nano-emulsification (generation of the nanodroplets).

Despite this, one would expect that the absence of any heating step would lead to a relatively constant ratio among EO phytochemicals.Some points can thereforebe considered. It is well-known that diffusion of EO components triggers series of phenomena that can induce an antagonistic mechanism of destabilization (Ostwald ripening) of the opposed force stabilization (compositional ripening), both being responsible for further differences in the distribution of the phytochemicals between the internal/external phases [19] and, therefore, affecting their ratio. If we hypothesize that this behavior happens concomitantly to the generation of the nanodroplets during the titration process of nano-emulsification, and considering that whenEO compounds are located at the external aqueous phase they are more susceptible to volatilization, it can be expected to facilitate a loss.

In fact, even some of the components that may be used in nano-emulsions generate exotermic reactions (e.g., dimethylsulfoxide/water) [20], and even in the absence of an intended heating step (e.g.,the PIT method) some alteration of the ratios between EO phytochemicals might occur if evaporation of the more volatile ones happens after some external phase heating. In the cases of mixtures prepared with some alcohols and n-alkanes prepared with edible vegetable oil, enthalpies of mixing have beencalculated to be, respectively, strongly and slighly endothermic [21]. However, to the best of our knowledge, mixtures of endogenous constituents of EOs (terpenes), or EOs/co-surfactant(s)/non-ionic surfactant(s)/water, of interest inthe use of colloids due to the notable impairments for industry resulting from low-energy methods, have not been extensively investigated. Therefore, we believe that, despite non-heating methods beingcapable of maintaining the fingerprint of an EO, it is important to evaluate the EO’sprofile after the preparation of the nano-emulsion as well as the ratio of the chemical markers, especially in the case of remarkable compounds, such as lemongrasses that contain high citral content.

Lu and co-workers [22] have also developed citral-based nano-emulsions but using a high-energy method and Span 85^®®^ and Brij 97^®®^ as surfactants. They verified that the HLB value influenced the size of the droplet size in an inverse manner. While the droplet size at HLB = 9 was 126 nm, it practically increased twofold at HLB = 6. One would expect that EOs would have components that are “more hydrophilic” than the fatty acids in conventional oils constituted by non-secondary metabolites (e.g., olive oil). Therefore, it is common to see EO-based nano-emulsions even when developed with pure hydrophilic non-ionic surfactants, such as polysorbate 20 or polysorbate 80. Despite the finding that compositional ripening may trigger stabilization [19], it is well-established that addition of a lipophilic co-surfactant contributes to stabilization of nano-droplets, especially if a chemically similar pair of surfactants is used. Inthis context, the use of a pair of non-ionic surfactants in the present study proved to be a valuable strategy to obtain fine droplets of citral-rich *P. elongata* in the EO-based nano-emulsions.

Rao and McClements [19] hypothesized that abundant terpenes with high water solubility, defined as those that have a value for this parameter higher than 5 mg/L, would be more soluble in the external phase and that two situations might thus occur: (i) release of these “more hydrophilic” compounds and, therefore, enrichment of some droplets in “less hydrophilic terpenes”, leading to stabilization through compositional ripening; or (ii) conventional destabilization through Ostwald ripening. By studying the nano-emulsification of lemon oil, they observed that compounds with low water solubility, which can be also understood as high logP (>4.3), were stabilizing the system through a “Compositional ripening effect”. Therefore, considering the high citral content of this EO, further investigations should be performed to better elucidate the absence of multiple droplet populations, as well to complete the quantification of citral during storage and take into account droplet maintenance over a longer period with a wide range of non-ionic surfactant pairs. However, for the aims of the present study, it was sufficient to observe that it was possible to successfully generate fine nanodroplets that reached equilibria during the storage period analyzed, narrowing the curve of size distribution and avoiding phase separation.

Citral-based nano-emulsions, prepared with 1% terpene and polysorbate 80, were generated by ultrasound and analyzed at days 0 and 60. The droplet sizes were respectively 34 nm and 324 nm, while the PdIs were 0.294 and 0.1. The macroscopic analysis of these nano-emulsions showed that, from day 0 to day 15 (25 °C), they presented a clear and stable appearance, before starting to flocculate and presenting a milky appearance after 30 of storage [23].

Arnon-Rips and co-workers [24] developed a coarse emulsion through magnetic stirring using citral (5%) and sunflower oil (1:1), polysorbate 80 and double-distilled water. A nano-emulsion was obtained by submitting this coarse emulsion to ultra-sonication, the initial droplet size and PdI of the coarse emulsion being reduced from 4 µm and 0.948 to 400–500 nm and 0.375, respectively, after the nanosizing. Concerning the stability, the coarse emulsion showed phase separation after 1 h at room temperature, while the nano-emulsion maintained stability for weeks.

## 4. Materials and Methods

### 4.1. Essential Oil

#### 4.1.1. Obtainment by Hydrodistillation

*Pectis elongata* was collected from a local farmer at the green rural area (S 02°27.8′143″–W 54°41.31′646″) of Santarém (Pará, Brazil).

The essential oil was extracted inthe Laboratory of Bioprospection and Experimental Biology (LabBBEx) of the Federal University of Oeste do Pará (UFOPA). It was obtained by hydrodistillation of the dried aerial parts of *P. elongata* on a Clevenger-type apparatus overa period of 2 h. Prior to the chemical analysis and nano-emulsification, the EO was dried over anhydrous sodium sulphate and stored protected from light.

#### 4.1.2. Characterization by GasChromatography (GC)

The EO was directly solubilized in hexane. An Rxi-5ms (5% diphenydilmethylpolysiloxane) fused silica capillary column (Restek Corporation, Bellefonte, PA, United States); 30 m × 0.25 mm i.d., 0.25 µm phase thickness) was used. The experimental conditions were as follows: injection mode was split (split ratio = 1:20); the injector temperature was 250 °C; the carrier gas was helium (99.995%); the flow rate was 1.0 mL/min; the oven temperature (column temperature) was 60–240 °C at 3 °C/min, ending with an isothermal at this final temperature for 10 min; the electron ionization mode was set at 70 eV; the ion source temperature was 200 °C; and the interface temperature was 250 °C. The mass spectra were recorded with an automatic scan of 0.3 s and mass fragments at a mass range of 35–400 *m*/*z*. The software GCMS-Solution (Shimadzu Corporation, Tokyo, Japan) was used for interpretation of the spectra and comparison to authentic libraries (Adams and FFNSC2); specifically, the retention index and the fragmentation pattern used for the identification of the compounds [14]. The relative percentage of the compounds was obtained with a normalization method using a GC 2010 Ultra System (Shimadzu Corporation, Tokyo, Japan) gas chromatograph coupled to a flame ionization detector. The experimental conditions were the same as previously described except the carrier gas, which here was nitrogen.

### 4.2. Nano-Emulsion

#### 4.2.1. Nano-Emulsification Process

The nano-emulsions were prepared with a low-energy, non-heating method and were composed of 5% (*w*/*w*) EO, 5% (*w*/*w*) non-ionic surfactants and 90% (*w*/*w*) deionized water. The oily phase (OP) was constituted by the non-ionic surfactants (polysorbate 80 and sorbitan trioleate) and the EO. Prior to the obtainment of the OP, its components were pooled together and vigorously mixed in a vortex stirrer. The surfactant-to-oil ratio (SOR) was 1, therefore indicating that the amount of the blend of non-ionic surfactants was the same as forthe EO used in the OP. The amounts of polysorbate 80 and sorbitan trioleate were calculated in order to achieve a blend with a hydrophilic–lipophilic balance (HLB) value of 13. Then, deionized water was added dropwise under vortex stirring and the bluish reflection associated with the Tyndall effect was considered indicative of the generation of nano-emulsion. The nano-emulsification was undertaken in triplicate.

##### Gas Chromatography Profile

In order to evaluate whether the nano-emulsification affected the overall phytochemical profile of the EO in the nano-emulsion immediately after the process of its preparation, the nano-emulsion was subjected to gas chromatography analysis. It was partitioned with hexane (1:3, *v*/*v*) and the mixture was vigorously mixed and left to equilibrate for 30 min. This procedure was repeated 10 times. The organic layer was collected for injection with a GCMS-QP2010 Ultra chromatograph (Shimadzu Corporation, Tokyo, Japan) under the same experimental conditions described for the characterization of the EO.

##### Citral Ratio

The citral ratio (CR) was calculated in order to verify whether the nano-emulsification affected the ratio between the geranial and neral immediately after the process of its preparation. It was expressed as a function of the percentages of geranial/neral in the nano-emulsions, as follows:

CR = %geranial/%neral

%geranial= (Ageranial × 100)/Atotal

%neral = (Aneral × 100)/Atotal

where Ageranial is the area of geranial obtained by peak normalization, Aneral is the area of neral obtained by peak normalization and Atotal is the sum of Ageranial and Aneral.

#### 4.2.2. Physical Characterization

The droplet size distribution of the nano-emulsions was performed using dynamic light scattering analysis on a Malvern ZetasizerNanoZS (Malvern, UK) [14,17] with the following specifications: laser source: 10 mW, λ 632.8 nm; scattering angle for size measurements: 173°. The results were given as the cumulant (z-average) size and the polydispersity index (PdI) for the size distribution of the droplets was expressed as the mean ± SD values. The zeta potential was also measured on the same equipment and expressed as mV values.

##### Droplet Growth (DG)

The behavior of the nano-emulsions was evaluated in term of the droplet growth (expressed as the percentage of the droplet size increase) [25] within a period of storage and was calculated as follows:

DG = [(Sizet2 − Sizet1)/Sizet1] × 100,(1)

where Sizet1 is the average size in the lower limit and Sizet2 is the average size in the upper limit of the t1 → t2 range of the DG calculation.

## 5. Conclusions

Low logP values and relatively high water solubility highlight the peculiarity of citral and may trigger the destabilization of nano-emulsions rich in the essential oil that is abundant in these terpenes. Therefore, we described a facile, non-heating, low-energy method for a lemongrass that can be used as a prototype for further industrial applications of high-citral-based nano-emulsions, especially for incorporation of this bioactive mixture of monoterpenes in cosmetics and pharmaceuticals or for citral-rich essential oils in phytocosmetics and phytotherapics.

## Figures and Tables

**Table 1:**
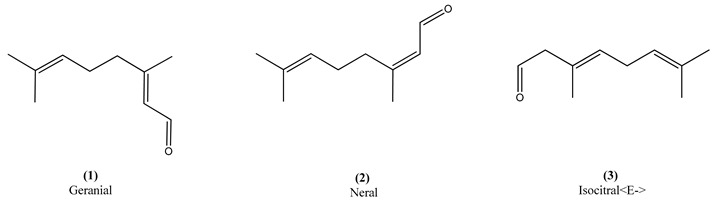
Gas chromatograph analysis of the *Pectis elongata* essential oil (EO) before and after its nano-emulsification (NE1,2,3 = independent replicates). RI_lit_—retention index from the literature (Adams); RI_exp_—retention index (experimental); n.d.—not determined.

RI_lit_	Compound	EO		NE_1_		NE_2_		NE_3_	
		Area (%)	RI_exp_	Area (%)	RI_exp_	Area (%)	RI_exp_	Area (%)	RI_exp_
936	Heptanone<5-Methyl-3->	0.12	941	n.d.	n.d.	n.d.	n.d.	n.d.	n.d.
958	Heptan-2-ol<6-Methyl-	0.13	950	n.d.	n.d.	n.d.	n.d.	n.d.	n.d.
981	Hepten-2-one <6-Methyl-5->	0.38	984	0.59	984	0.63	984	0.57	984
1024	Limonene	0.45	1027	0.55	1028	0.52	1028	0.52	1028
1085	Cyclohexanedione <3-Methyl-1,2->	0.17	1090	n.d.	n.d.	n.d.	n.d.	n.d.	n.d.
1095	Linalool	0.32	1099	0.52	1099	0.56	1099	0.52	1099
1140	Isocitral<Exo->	0.12	1143	0.16	1143	0.17	1143	0.16	1143
1160	Isocitral<Z->	0.73	1162	0.59	1163	0.57	1163	0.55	1163
1177	Isocitral<E->	1.41	1181	1.61	1181	1.55	1181	1.49	1181
1227	Nerol	0.46	1227	0.71	1229	0.8	1229	0.76	1229
1235	Neral	39.35	1242	41.89	1245	41.48	1245	40.91	1245
1249	Geraniol	0.77	1253	0.79	1256	0.66	1255	0.6	1255
1268	Geranial	50.76	1272	49.92	1276	50.37	1276	51.25	1276
1288	Lavandulyl Acetate	0.2	1278	n.d.	n.d.	n.d.	n.d.	n.d	n.d.
1290	Tridecene <1->	1.31	1290	n.d.	n.d.	n.d.	n.d.	n.d	n.d.
n.d.	Geranic acid	n.d.	n.d.	0.14	1356	0.19	1356	0.16	1356
1365	Undecenol<2E->	0.74	1371	n.d.	n.d.	n.d.	n.d.	n.d	n.d.
1389	Elemene<Beta->	0.27	1390	n.d.	n.d.	n.d.	n.d.	n.d	n.d.
1452	Humulene <Alpha->	0.67	1452	0.19	1452	0.16	1452	0.17	1452
1608	Humulene Epoxide II	0.23	1606	0.23	1607	0.21	1607	0.23	1607

**Table 2:** Dynamic light scattering (droplet size and polydispersity index) and zeta potential values after preparation (day 0) and after storage of the nano-emulsions prepared with the essential oil of *Pectis elongata*.

Nano-Emulsion (Replicate 1)							
	Day 0	Day 1	Day 7	Day 14	Day 30	Day 60	Day 90
Size (nm)	119.8 ± 0.4509	117.4 ± 0.7638	114.2 ± 1.012	117.5 ± 0.4163	138.9 ± 0.1732	180.4 ± 1.286	217.7 ± 1.900
Polydispersity index	0.217 ± 0.018	0.221 ± 0.005	0.201 ± 0.009	0.158 ± 0.006	0.119 ± 0.013	0.086 ± 0.012	0.083 ±
Zeta potential	−3.66 ± 0.0902	−2.17 ± 0.148	−2.53 ± 0.127	−1.61 ± 0.142	−1.31 ± 0.0702	−8.05 ± 0.230	−0.279 ± 0.406
**Nano-Emulsion (Replicate 2)**							
	Day 0	Day 1	Day 7	Day 14	Day 30	Day 60	Day 90
Size (nm)	113.4 ± 1.015	111.7 ± 0.6658	109.0 ± 0.3786	111.0 ± 0.8386	125.0 ± 0.4041	155.4 ± 1.834	180.5 ± 1.604
Polydispersity index	0.223 ± 0.018	0.209 ± 0.011	0.193 ± 0.006	0.171 ± 0.005	0.119 ± 0.016	0.091 ± 0.007	0.089 ± 0.026
Zeta potential	−2.34 ± 0.366	−1.44 ± 0.369	−3.04 ± 0.215	−1.77 ± 0.0757	−1.63 ± 0.429	−3.11 ± 0.527	−0.0173 ± 0.266
**Nano-Emulsion (Replicate 3)**							
	Day 0	Day 1	Day 7	Day 14	Day 30	Day 60	Day 90
Size (nm)	115.8 ± 0.1155	114.2 ± 1.350	114.7 ± 0.1155	111.3 ± 0.1528	123.1 ± 1.015	155.0 ± 1.082	183.4 ± 1.058
Polydispersity index	0.204 ± 0.011	0.223 ± 0.017	0.226 ± 0.018	0.176 ± 0.008	0.120 ± 0.014	0.087 ± 0.022	0.074 ± 0.015
Zeta potential	−2.38 ± 0.123	−2.03 ± 0.0529	−4.40 ± 0.0896	−2.42 ± 0.199	−2.36 ± 0.0252	−3.42 ± 0.173	−1.69 ± 0.383
**Average**							
	Day 0	Day 1	Day 7	Day 14	Day 30	Day 60	Day 90
Size (nm)	116.4 ± 2.868	114.4 ± 2.612	112.6 ± 2.820	113.3 ± 3.180	129.0 ± 7.484	163.6 ± 12.64	193.9 ± 19.96
Polydispersity index	0.215 ± 0.016	0.218 ± 0.012	0.207 ± 0.018	0.168 ± 0.010	0.119 ± 0.012	0.088 ± 0.013	0.082 ± 0.018
Zeta potential	−2.80 ± 0.681	−1.88 ± 0.390	−3.32 ± 0.847	−1.93 ± 0.391	−1.77 ± 0.515	−4.86 ± 2.41	−0.662 ± 0.838

## Sample Availability

Samples of the essential oil are available from the authors.
